# The effect of ongoing feedback on physical activity levels following an exercise intervention in older adults: a randomised controlled trial protocol

**DOI:** 10.1186/s13102-016-0066-5

**Published:** 2017-01-10

**Authors:** Katie-Jane Brickwood, Stuart T. Smith, Greig Watson, Andrew D. Williams

**Affiliations:** 1 School of Health Sciences, University of Tasmania, Locked Bag 1322, TAS 7250 Launceston, Australia; 2 Primary Health Tasmania, TAS 7250 Launceston, Australia; 3 Coffs Harbor Campus, Southern Cross University, NSW 2450 Coffs Harbour, Australia

**Keywords:** Physical activity maintenance, Behaviour change, Remote feedback

## Abstract

**Background:**

Physical inactivity ranks as a major contributing factor in the development and progression of chronic disease. Lifestyle interventions reduce the progression of chronic disease, however, compliance decreases over time and health effects only persist as long as the new lifestyle is maintained. Telephone counselling (TC) is an effective way to provide individuals with ongoing support to maintain lifestyle changes. Remote physical activity monitoring and feedback (RAMF) via interactive technologies such as activity trackers and smartphones may be a cost-effective alternative to TC, however, this comparison has not been made. This study, therefore, aims to determine the effect of ongoing feedback (TC vs. RAMF) on the maintenance of physical activity following a 12-week individualised lifestyle program, and the effect of this on health risk factors and health services usage.

**Methods and design:**

A randomised controlled trial with a parallel groups design. A total of 150 adults (≥60 years) who participate in a 12-week face-to-face individualised lifestyle program will be randomised to twelve months of RAMF (*n* = 50), TC (*n* = 50), or usual care (*n* = 50). Participants randomised to RAMF will use a smartphone activity tracker app, synced to a wrist worn activity tracker, to provide them with automated feedback regarding compliance to prescribed activity targets. Telephone counselling involves a follow-up phone call every fortnight for the first three months and a monthly call for the remaining nine months of the follow-up period.

The primary outcome measures are physical activity compliance (accelerometry and Active Australia survey). Secondary outcome measures include cardiorespiratory fitness, muscle strength, dynamic balance, quality of life, blood pressure, body composition, and health services usage. Measures will be made before and after the individualised lifestyle program, and at three, six and twelve months during the intervention.

**Discussion:**

The results of this study will help to determine the efficacy of RAMF devices on compliance to prescribed physical activity compared to the current gold standard of TC. If the remote monitoring proves effective, it may provide a cost efficient alternative method of assisting maintenance of behaviour change from lifestyle interventions.

**Trial registration:**

ACTRN12615001104549. Retrospectively Registered 20/10/2015.

## Background

### Ageing and low physical activity levels

There is a substantial body of research which supports the benefits of regular physical activity for health (eg. [1]). Participation in physical activity has been shown to decrease the risk of morbidity and mortality, and loss of independence in older adults and plays an important role in maintaining functional capacity [2]. However, despite the well-known benefits of regular participation in physical activity, physical inactivity in the industrialised world ranks as a major contributing factor in ill-health. Worldwide 31% of the adult population are not meeting the minimum recommendations for physical activity to perpetuate and maintain health in order to prevent disease [3]. Australia’s population over the age of 65 has increased from 1.1 M (9%) in 1973 to 3.3 M (14%) in 2013 [4], and along with this growing population of elderly citizens the number of cases of degenerative and lifestyle diseases has also increased [1]. The highest levels of physical inactivity are also evident in older adults with 18% of Australians aged 40–65 reporting no physical activity [1].

### Benefits of exercise

Strength and/or endurance training activities have been shown to prevent and manage heart failure, diabetes, depression, arthritis and many other conditions that effect older adults [5–7]. However, an issue with much of the existing research is that it has generally involved laboratory-based exercise interventions which have been tightly controlled and attract the most motivated participants. Consequently, while these studies have been helpful in describing many benefits of exercise for a range of chronic conditions, they do not readily translate to interventions that are achievable in clinical practice.

### Lifestyle interventions, issues with ongoing compliance

The use of individualised lifestyle intervention programs may provide a means of addressing the challenges associated with including exercise as a treatment in chronic conditions. Research has reported that lifestyle intervention programs are effective in reducing the progression of chronic disease [8, 9]. While lifestyle interventions are effective in managing chronic disease, there are still issues with ongoing compliance to the altered lifestyle declining over time and health effects only persist as long as the new lifestyle is maintained [10]. Telephone counselling has been shown to be a feasible and cost effective way to provide individuals with ongoing support to maintain lifestyle changes [11, 12]. Review of the literature has shown that interventions lasting greater than 6 months and those with a higher number of calls produce the most favourable outcomes [12].

### Role of new technologies

One method for increasing enjoyment and objectively tracking adherence to exercise programs involves the use of interactive technologies like physical activity trackers. Consumer driven forces for new ways to measure physical activity have led to the development of sophisticated inertial sensing devices for measuring movement of the human body. Until recently, such technology could only be found in expensive and dedicated laboratory facilities.

Devices are now at a price point that it is possible to relatively inexpensively deploy feedback technologies for use in individualized physical activity programs. The use of mobile phones/computers, combined with ubiquitous tracking technologies and management applications, has enabled individuals to better understand their personal health. As smart phones have become more common, two parallel developments have created an easier to use interface than ever before – the large touch screen; and significantly more complex functionality in accelerometers, compasses, GPS and other sensing devices. Additionally, third party creators of specialist sensing devices are now supplying the feedback from these sensors into these smartphones.

Globally, there has been in increase in apps which are designed to improve health through better lifestyle choice, including tracking food intake, tracking activity, encourage healthy food and activity replacements, retaining mental flexibility and support for giving up smoking. Personalised social media-based portals could facilitate motivation for exercise progression through social support [13], including peer- and professional-support [14], and promote health behaviour change [15]. Paired with mobile computing technology, it will be possible to extend current telephone health-coaching concepts to these more automated platforms potentially resulting in greater ongoing compliance to individualized lifestyle programs and resultant improved health outcomes. Emerging technologies which include activity trackers that can provide real time feedback on activity levels and remote social interaction may provide an alternative means of continuing to motivate participants beyond the completion of a formal exercise/lifestyle program. To date however, there is limited research examining the effect of such technologies on the maintenance of activity levels, particularly in older adults.

## Objectives

The study aims to:

1. determine the effect of different modalities of ongoing feedback regarding physical activity on the maintenance of activity levels post completion of a 12-week individualised lifestyle program and;
2. The effect of ongoing physical activity levels on a range of health risk factors and health services usage.

The study will measure and monitor the activity levels of participants during the community based individualised lifestyle program and for 12 months post completion of the program. Participants will be randomly assigned to 1) usual care in the form of physical activity questionnaires plus assessment at 3, 6 and 12 months post program, 2) provided with ongoing feedback through a wrist worn activity tracker and smartphone for the duration of the follow up period and assessment at 3, 6 and 12 months post program or 3) provided with fortnightly to monthly telephone calls for the duration of the follow up period and assessment at 3, 6 and 12 months post program. Follow up assessment at 3, 6 and 12 months will include the collection of physiological variables, functional testing, physical activity and quality of life questionnaires, and health services usage data.

## Methods

### Study protocol

**Fig. 1:**
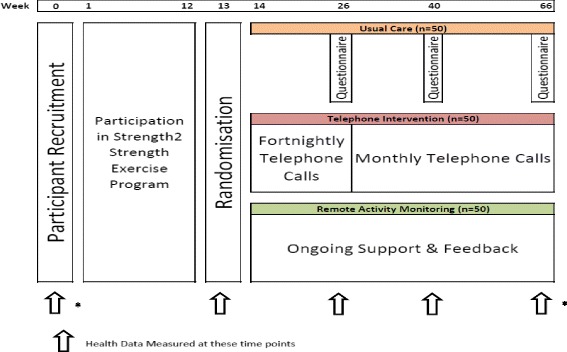
Study Design showing time points for data collection, lifestyle intervention and the three methods of feedback

The study involves a parallel group randomised control design to evaluate the potential for activity trackers (Jawbone; Jawbone, Inc. 99 Rhode Island Street, San Francisco, CA 94103) to be used to provide regular feedback to assist with maintenance of new exercise behaviours following a12-week face to face individualised exercise lifestyle program run by exercise physiologists (Strength2Strength Tasmania Exercise Treatment Initiative). 150 participants who have been referred to the individualised lifestyle program will be recruited for this study. Recruitment will occur through personal invitation at the time of their initial assessment for the lifestyle program. Participants will undergo a suite of tests for variables associated with physical function and clinical prognosis and the beginning and end of the face to face program and at 3, 6 and 12 months of follow-up (Fig. 1). Participants randomised to the activity tracker group will be compared to those who receive usual care and to those who receive telephone counselling, the current best practice for maintenance of lifestyle change.

### Ethical considerations

The study has been approved by the Tasmania Health and Medical Human Research Ethics Committee. Annual progress reports will be made to the ethics committee and they will be notified of any adverse events promptly. The trial has been registered with the Australian New Zealand Clinical trials Registry (ACTRN12615001104549, http://www.anzctr.org.au/default.aspx). Annual progress reports will be provided to the ethics committee and any adverse outcomes will be immediately reported. All data collected will be stored as paper files in a locked filing cabinet and/or electronic files which will be stored on a password protected computer. Only investigators directly associated with the study will have access to these files. Following the completion of the study, participants will have access to their data.

### Selection of participants

Inclusion Criteria are individuals referred to the Strength2Strength (S2S) Tasmania Exercise Treatment Initiative. The S2S Tasmania Exercise Treatment Initiative is an individually tailored 12-week exercise lifestyle program that is led by Accredited Exercise Physiologists and targeted at individuals who are:

- elderly, frail and aged
- are at risk of developing chronic disease
- have a chronic medical condition including neurodegenerative disease, cardiovascular disease and conditions, chronic obstructive pulmonary disease (COPD), type 2 diabetes, muscular and/or skeletal conditions, mobility or gait issues and those receiving steroid treatment.

Due to some of the outcome variables from S2S being utilised in the research, participants will be recruited as they commence S2S. The proposed research will investigate the ongoing engagement of S2S participants once they have exited that program. Some of the outcome variables from the S2S program will be utilised in the proposed research but only for those participants who consent to participate in the research study. The reason for use of these outcome variables is to inform of changes in health and physical activity status that occurred during S2S as these changes may impact on how physical activity levels and health outcomes change during the follow up (intervention) period. Data will be collected from S2S site in Launceston, Tasmania, Australia only.

Exclusion criteria: Individuals who choose not to participate in the S2S program will not be considered. Anyone presenting with an unstable medical condition preventing them from participating in regular physical activity; or individuals with neurological conditions, or limited understanding of English which prevent them from meeting the self-reporting requirements of the study will also be excluded.

### Randomisation

Participants who enrol in the study will undergo baseline testing and the 12 week S2S program before being randomised to one of the three intervention groups. Randomisation of participants to intervention groups will be performed at the end of the S2S endpoint testing session. Randomisation will be performed using computer generated blocks of fifteen by a third person not directly involved in the study and will be recorded in sealed opaque envelopes. Each envelope will be opened sequentially at the endpoint S2S endpoint testing session to reveal the allocated intervention. In the event that there are multiple participants from the same household they will be randomised to the same group to maintain the integrity of the intervention. All participants will be requested to avoid the use of any physical monitoring devices other than those directly provided by the researchers for the duration of the study.

## Interventions

### Usual care group

**Fig. 2:**
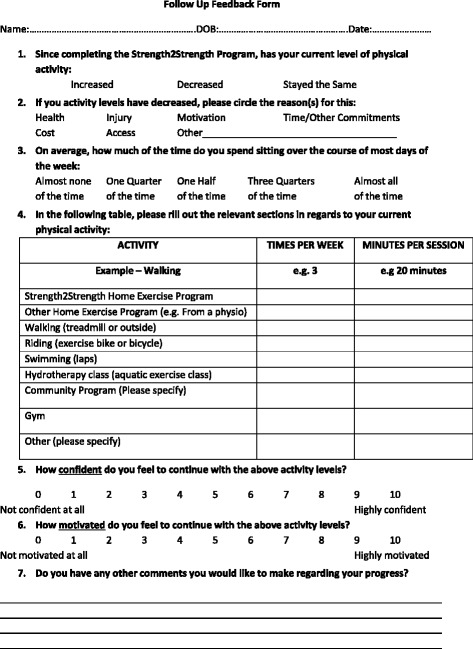
Usual Care Follow Up Questionnaire administered at 3, 6 and 12 months’ post program

The usual care group (*n* = 50) will receive the usual care offered to participants who complete the 12-week Strength2Strength program. Follow up surveys are sent to participants at 3, 6 and 12 months’ post program to determine their current level of self-reported physical activity and sitting time (Fig. 2). Participants are also asked if they feel their activity levels have increased, decreased or stayed the same, reasons for a decrease in activity levels and how confident and motivated they are to continue to be physically active. Participants will attend assessments at 3, 6 and 12 months’ post program completion for collection of physiological variables, functional testing, physical activity and quality of life questionnaires, and health services usage data.

### Remote physical activity monitoring

Participants randomised to the remote physical activity monitoring group (*n* = 50) will receive a Jawbone activity tracker at the end of the 12 week S2S program. They will be requested to wear the Jawbone on their wrist, or ankle if the wrist is not a suitable option, for the duration of the 12 month follow up period. The activity tracker will be set up to provide feedback, via vibrotactile alert, to participants on their compliance to their individual prescribed activity levels (50 & 100% of prescribed daily activity). These levels will be prescribed by the exercise physiologists as part of the S2S program. In addition to the feedback achieved while wearing the device, participants will be requested to synchronise their Jawbone device to the study database at the end of each day using a mobile internet link they will be provided with as part of the study. The daily synchronisation will also serve to recharge the Jawbone device for the following day’s use. In the event that a participant fails to synchronise the device at the end of the day they will be sent an automated text message reminding them of the need to recharge their device regularly and the synchronisation and recharge will occur when they next connect. As part of the daily synchronisation participants will receive further feedback regarding their overall progress during the intervention. This will include feedback regarding progress towards daily and weekly physical activity targets, general health tips and remote feedback from the Exercise Physiologists regarding ongoing compliance to the individualised exercise program. Participants will attend assessments at 3, 6 and 12 months post program completion for collection of physiological variables, functional testing, physical activity and quality of life questionnaires, and health services usage data.

### Telephone counselling

**Fig. 3:**
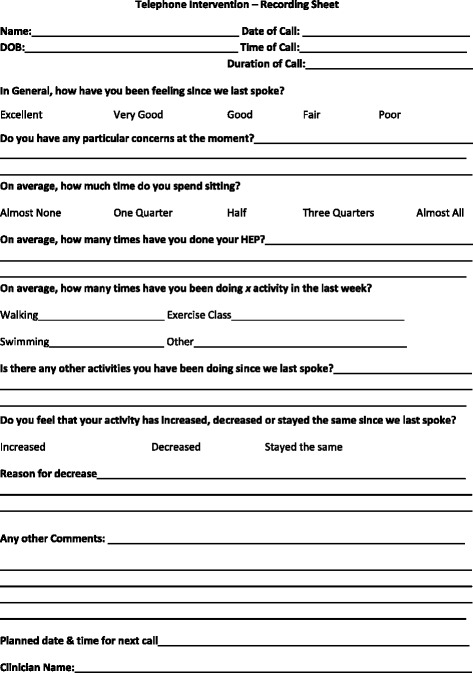
Telephone consultation recording sheet

Following the completion of the 12-week Strength2Strength program, participants (*n* = 50) will receive a follow up phone call once a fortnight for the first 3 months of the follow up period, and once a month for the remaining 9 months of the follow up period from the Exercise Physiologists. Participants will be asked to self-report on how they feel they are going with their activity levels and compliance to the home based program and if there are any issues that they feel are preventing them from being more physically active (Fig. 3). Participants will be provided assistance for any specific issues reported. Participants will attend assessments at 3, 6 and 12 months post program completion for collection of physiological variables, functional testing, physical activity and quality of life questionnaires, and health services usage data.

### Outcome measures

Outcome data pertaining to physical function, quality of life, physical activity levels, and markers of clinical prognosis (BMI, waist circumference, brachial blood pressure) will be collected at the start and end of the 12 week S2S program and again at 3, 6 and 12 months of follow up after the participants have been randomised to one of the 3 interventions. All outcome measures will be collected for all three treatment groups at all time-points.

### Physical activity measurement

Physical activity will be measured using two tools (a physical activity tracker and a questionnaire). Physical activity will be measured for a period of seven days at each time point through the use of ActivPal activity trackers. These will be fitted and worn day and night for the entire seven-day period with a minimum of five days’ worth of valid data required for inclusion at each assessment time. These trackers are reliable and valid [16, 17] for physical activity monitoring. Data obtained from the ActivPal activity trackers will be the primary measure of physical activity and expected changes to data obtained from these trackers form the basis of the sample size calculation for this study.

The physical activity questionnaire that will be used is the Active Australia questionnaire [18]. It will be included as a back-up activity monitor and to assess changes in time spent in different types of activities. Participants will be requested to include any exercise performed in the previous week in their response to the questionnaire. The survey measures frequency, intensity and duration of incidental and/or intentional physical activity in the week prior to the time of testing. The total time spent in each activity will be multiplied by an intensity value and used to calculate participants’ weekly physical activity in MET.min^−1^. This survey is valid and reliable [19, 20] and has previously been used to measure changes in physical activity in chronic disease populations [21].

### Motivation for exercise

The self-regulation Questionnaire for exercise (SRQ-E) is a theoretically derived questionnaire developed for use in school settings but which has since been validated in diverse health settings [22]. The SRQ-E gathers information on why a person chooses to exercise regularly and assesses the degree to which individuals are autonomous in exercise behaviour.

### Self-reported physical function (quality of life)

Self-perceived physical function will be evaluated using the Medical Outcomes Short-Form 36-Item Health Survey (SF-36). This survey includes 8 independent scales and assesses physical and mental dimensions of health. It is validated and has been widely used to measure quality of life in a range of chronic disease populations [21, 23, 24].

### Physical function

The six-minute walk test (6MWT) or Modified Shuttle Walk Test (MSWT) will be used as the measure of physical function. Both tests require minimal equipment and are quick and easy to conduct. The 6MWT requires participants to walk as far as possible along a short measured course during the six minutes. They will be permitted to stop and rest if needed and will receive regular encouragement throughout the test. The MSWT is an incremental walk test requiring participants to walk along a short measured course while keeping pace with a recording. Both the 6MWT [25] and MSWT [26] are reliable and valid measures of physical function in patients with metabolic disease.

### Muscle strength

Strength will be measured using the ten times sit to stand (TTSTS). TTSTS is a functional lower body strength measure that relates to ability to perform activities of daily living and consequently is relevant to wider health and quality of life outcomes. It is a reliable and valid measure that is widely used in clinical practice [27].

### Dynamic balance and mobility

Dynamic balance and mobility will be measured using the timed up and go (TUG). This test involves the client standing up from an armless chair and walking around a cone placed 2.44 m in front of the chair, returning to the chair and sitting down. It is a reliable and valid measure of dynamic balance and is widely used in clinical practice [28].

### Body composition

Three indicators of body composition will be measured. These are body mass index, waist circumference and percent body fat. These measures are simple and easy to attain and have been widely used as indicators of cardiovascular disease (CVD) [29] and mortality [30] risk in large scale population studies. Body Mass Index (BMI) will be calculated from height and body weight. Waist circumference will be measured at the narrowest point between the base of the rib cage and the iliac crest as per standard methods. Body fat percent will be assessed through the use of Bio-Impedance Analysis scales (Tanita BC-1000; Tanita Corp; Tokyo, Japan). BIA has previously been shown to be a reliable and reproducible method for determining body composition [31, 32].

### Blood pressure

Brachial blood pressure will be measured by sphygmomanometry using standardised techniques after five minutes of seated rest.

### Health Service Usage

**Fig. 4:**
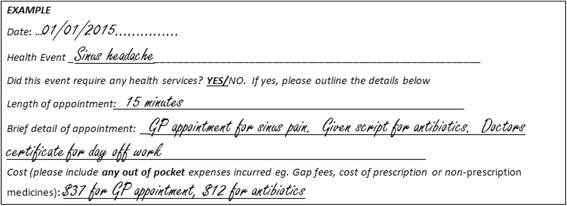
Example Health Service Usage Diary entry

Retrospective (covering before and during the study) information about medications (Medical Benefits Scheme (MBS) and Pharmaceutical Benefits Scheme (PBS) items of service from the Australian Department of Human Services, hospitalisations (Department of Health and Human Services Clinical Costing Dataset [electronic inpatient separation summary] for costing’s and morbidity) and mortality (AIHW National Death Register) will be collected. Out-of-pocket expenses, incidental and non-billable health professional encounters, and work missed through ill health will be assessed by health diaries to be maintained by each patient over the intervention and follow up periods (Fig. 4).

### Adherence to the intervention

Participant adherence to the treatment interventions will be assessed through records of the proportion of intervention days that participants randomised to the remote physical activity monitoring group wear their activity monitor. Records will also be kept and reported regarding the the proportion of telephone calls successfully delivered to participants in the telephone counselling group as well as the length and content of these calls.

### Sample size calculation

The 12 week S2S intervention resulted in a mean increase of 450 MET.min of physical activity per week with a SD of 1.5x the change. Sample size was calculated based on a predicted maintenance of 100% of additional physical activity in the feedback groups and decrease of 50% of additional physical activity in the usual care group over the 12-month intervention (Mean Difference = 225 Met.min/week) with a SD of the change of 350 MET.min/week (1.5x the mean change in line with S2S data). STATA 12 (Statistical data analysis; Stata Corp; College Station; Texas, USA) was used to calculate the sample size on the basis of a mean difference of 225 MET.min/week with a SD of the change of 350 MET.min/week, a power of 80% and an alpha level of 0.05 using the “sampsi 0 225, sd (350) p (0.8) a (0.05)” command. The sample size calculation indicated that at least 38 participants were required per group. To allow for potential withdrawals 50 participants per group will be recruited.

## Data analysis

All statistical analyses will be performed using STATA statistical software (STATA 13; Statistical data analysis; Stata Corp; College Station; Texas, USA). Baseline and descriptive data between the groups will be analysed with one-way ANOVA. Binary categorical data will be analysed for variability between the groups using logistic regression. Parametric longitudinal data will be analysed using mixed effects linear regression with unstructured covariance, corrected for repeated measures. In the event that assumptions of linear regression (heteroskedascity, skewness, kurtosis or non-linearity) are violated, those analyses will be repeated using repeated measures ordinal logistic regression. *P*-Values will be corrected where appropriate for multiple comparisons using the Holm test. Where participants do not return for follow up or there is missing data, this will be analysed on a last case brought forward basis.

## Discussion

The objective of this trial is to determine the effect of different modalities of ongoing feedback regarding physical activity on the maintenance of activity levels post completion of a 12-week individualised lifestyle program and; the effect of ongoing physical activity levels on a range of health risk factors and health services usage. Due to the ageing and increasingly inactive global population, sustainable and cost effective measures to assist older adults to maintain an increased level of activity need to be put in place. The effectiveness of telephone counselling on behaviour change has been well documented [11, 12] however it is time consuming and expensive when continued over a prolonged period of time. In contrast the effectiveness of remote feedback devices on compliance to prescribed physical activity have not been investigated. The results of this study will help to determine the efficacy of remote physical activity monitoring and feedback devices on compliance to prescribed physical activity compared to the current gold standard of telephone counselling. If the remote monitoring and feedback proves effective, it may provide a cost efficient alternative method of assisting maintenance of behaviour change from lifestyle interventions.
